# Improved Prognostic Staging in Endometrial Cancer: Clinical Impact of Aggressive Subtypes in a Multicenter Cohort

**DOI:** 10.3390/medsci14030414

**Published:** 2026-07-22

**Authors:** Tatiana Cuesta-Guardiola, Alicia Quirós, Pluvio Jesús Coronado Martín, Augusto Pereira Sánchez

**Affiliations:** 1 Department of Obstetrics and Gynecology, University Hospital of León, 24008 León, Spain; tcuestag@saludcastillayleon.es; 2 Department of Mathematics, University of León, 24008 León, Spain; alicia.quiros@unileon.es; 3 Department of Gynecologic Oncology, Department of Obstetrics and Gynecology, Hospital Clínico San Carlos de Madrid, 28040 Madrid, Spain; plujcoro@ucm.es; 4 Department of Gynecologic Oncology, Hospital Universitario Puerta de Hierro de Majadahonda, 28222 Madrid, Spain

**Keywords:** endometrial cancer, aggressive subtypes, survival, tumor classification, risk stratification

## Abstract

**Objectives**: Assessment of the impact on survival of endometrial carcinoma according to the 2009 FIGO (International Federation of Gynecology and Obstetrics) classification and the new FIGO 2023 classification highlighting the worse prognosis of the aggressive subtypes. **Methods**: This multicenter retrospective study included 1181 patients with endometrial cancer. Comprehensive clinical, pathological and treatment-related variables were collected. Primary outcomes included overall survival assessed through five-year follow-ups. Statistical analysis included comparative tests, Kaplan–Meier survival estimation, Cox proportional hazards models and ROC curves analysis to review prognostic accuracy. **Results**: Aggressive endometrial carcinoma (n = 353) showed significant worse overall survival compared with non-aggressive cases (35.7 versus 60 months). A novel classification based on FIGO 2023 was developed, integrating histological aggressiveness into a different stage and combining early non-aggressive stages in only one stage. While FIGO 2009 and 2023 classifications showed prognostic value, the new model improved risk stratification, clearly distinguishing high-risk groups. Multivariate analysis identified aggressive subtype, stage, age, diabetes, myometrial invasion and lymphovascular invasion as independent predictors. **Conclusions**: Aggressive histological subtype in endometrial cancer should carry greater prognostic weight in terms of survival and clinical management. Our findings support a potential shift in the current paradigm for these relatively rare but high-risk cases.

## 1. Introduction

Endometrial cancer is of great interest due to its increasing prevalence in developed countries [1,2], coupled with a significant mortality rate despite most cases being diagnosed at early stages [3]. The primary treatment is surgical, but many cases require additional adjuvant treatments such as radiotherapy or chemotherapy, which increase morbidity. It is crucial to select the optimal treatment for each patient to avoid unnecessary morbidity and ensure that no patient is undertreated, which would pose significant risks.

Due to the growing interest in the subject, new molecular and pathological characteristics of endometrial tumors have been identified in recent years. This has led to the publication of a new FIGO (International Federation of Gynecology and Obstetrics) classification for endometrial cancer in 2023 [4]. This classification aims to define prognostic groups more accurately and create a clinically relevant subclassification for treatment and future data collection.

The new classification includes a greater number of subgroups and places more emphasis on anatomical and pathological data, such as lymphovascular invasion [5] and histological groups considered high grade [6]. It also incorporates the molecular classification of endometrial cancer described by the Cancer Genome Atlas in 2013 [7]. This reorganization could significantly change patient expectations, treatment, and prognosis, but it is still uncertain whether it will be more beneficial compared to the previous classification.

As defined by the FIGO 2023 classification, non-aggressive histological types are composed of low-grade (grades 1 and 2) EECs. Aggressive histological types are composed of high-grade EECs (grade 3), serous, clear cell, undifferentiated, mixed, mesonephric-like, gastrointestinal mucinous-type carcinomas, and carcinosarcomas [4].

Given the new available evidence and the questioning of the need for a new classification based on scientific findings in gynecological cancer, the objective of this work is to assess the impact on survival and progression-free survival of women with endometrial carcinoma according to the FIGO 2009 [8] classification and the new FIGO 2023 classification [4] specially distinguishing the aggressive subtypes and the non-aggressive subtypes. Additionally, we will propose a new classification model in order to improve the prognosis and management of the disease, with possible changes in treatment in the future.

## 2. Materials and Methods

### 2.1. Study Design

This multicenter, observational, and retrospective study focuses on endometrial cancer (EC) patients who underwent diagnosis and primary treatment at Hospital Clínico San Carlos, Madrid; Hospital Universitario Puerta de Hierro Majadahonda, Madrid; and Complejo Asistencial Universitario, León, from 1990 to 2023. Data were retrieved from electronic records, and the study was reported following the STROBE guidelines [9].

All cases of apparent preoperative early FIGO stage endometrial cancer were surgically managed in specialized gynecologic oncology units by experienced surgeons, in accordance with international guidelines [10,11]. Each case was individually reviewed by the Institutional Multidisciplinary Tumor Board at the participating centers, where the surgical approach and indications for adjuvant treatment were determined on a case-by-case basis. Adjuvant therapy, including external beam radiotherapy, brachytherapy, and chemotherapy, was administered according to the current European Society of Gynecologic Oncology and the Spanish Society of Gynecology and Obstetrics guidelines at the moment of the diagnosis. Patient status at the last follow-up visit was recorded, with follow-up conducted at least every 6 months.

Five clinical variables were collected: age, body mass index (BMI), presence or absence of diabetes, presence or absence of high blood pressure, and parity status (nulliparous or multiparous). Anatomopathological data included histologic type, classified according to Bokhman’s criteria as type I (non-aggressive) or type II (aggressive). In the aggressive group, the specific histologic subtype was recorded (endometrioid carcinoma, serous carcinoma, clear cell adenocarcinoma, undifferentiated carcinoma, mixed carcinoma, or carcinosarcoma). Tumor grade (G1, G2, or G3), depth of myometrial invasion, LVSI, distinguishing “substantial” or “extensive” from “focal” or “absent”, cervical stromal invasion, adnexal involvement, and lymph node status were also evaluated.

Additional data recommended by the International Collaboration on Cancer Reporting (ICCR) 2021 [4] were reviewed, including the endometrial cancer stage according to FIGO 2009 and the new FIGO 2023 staging system. We accurately reviewed whether each case assessed initially staged with FIGO 2009 was upstaged, downstaged, or remained unchanged with the new FIGO 2023, and whether this change affected treatment. Treatment details, including surgical and chemo-radiotherapy reports, were reviewed, and side effects were categorized into mild, moderate, or severe according to the Clavien–Dindo classification [12]. The last follow-up date was recorded, detailing the outcome at five years: alive or dead. Overall survival was defined as the time from diagnosis to death (any cause).

Regarding reclassification, initial staging was based on the previous FIGO 2009 staging, and the new classification FIGO 2023 resulted in changes in some cases. Upshifting was considered a reclassification to a higher stage (e.g., from Stage I to Stage II, not considering a change from IA to IC), and downstaging was reclassification to a lower stage similarly.

### 2.2. Participants

**Figure 1:**
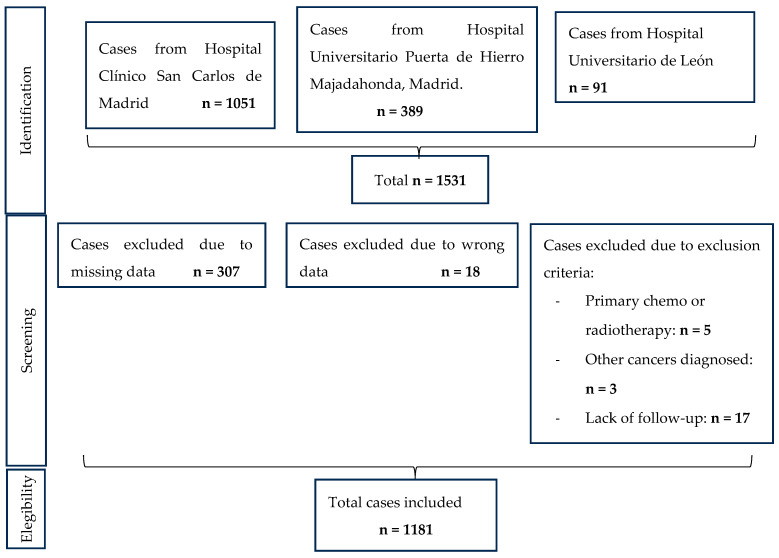
Flowchart of the search strategy, inclusion and exclusion criteria.

Exclusion criteria included cases treated with chemotherapy and/or radiotherapy as primary treatment, cases lacking pivotal pathological parameters such as lymphovascular space invasion (LVSI), tumor grade, or myometrial invasion, and patients without complete diagnosis or treatment details. Those diagnosed with uterine sarcomas or other cancers that could affect prognosis were excluded, as well as patients without at least 12 months of follow-up. In order to clarify the patient selection process carried out, Figure 1 is provided, including a flowchart that summarizes it.

### 2.3. Protocols

This study was conducted in compliance with the Declaration of Helsinki and approved by the respective Ethics Committees for Clinical Research of the participating centers. All participants were fully anonymized, and no treatment changes occurred due to participation in this study. Databases from the following studies were used: LICEAR [13] (ECRC nº 1003.381-E), LICERI [14] (ECRC nº 14.151-E), CERI [15] (ECRC nº 20.407-E), ROBOCOG [16] (ECRC nº 12.0035-E), and “Identification and characterization of endometrial carcinoma with tumor markers HE4 and CA125 in serum and endometrial tissue samples” [17] (ECRC nº 17104). Moreover, data from Hospital Puerta de Hierro Majadahonda Madrid were recruited with ECRC nº238/23.

### 2.4. Statistical Analyses

All statistical analyses were performed using Stata Statistical Software: Release 17 (StataCorp LLC, College Station, TX, USA), with two-sided *p*-values < 0.05 considered significant. Continuous variables were reported as mean ± standard deviation or median (25th–75th percentiles), depending on the normality of their distribution, assessed by the Kolmogorov–Smirnov test. Categorical variables were reported as count (percentage). Comparisons of categorical variables between case-control groups (aggressive vs. non-aggressive cases) were assessed using the Chi-Square test as applicable. For comparing continuous variables between aggressive/non-aggressive cases, the Student *t*-test was used if samples were normally distributed or had homogeneous variances; otherwise, the Mann–Whitney U test was used. Correlations between continuous variables were assessed using Pearson’s (r) for parametric variables or Spearman’s (ρ) rank correlation test for the opposite cases or when assumptions of normality were not met.

Extended long-term overall survival (OS) curves were estimated using the Kaplan–Meier method and compared using the log-rank test. OS analysis and outcome predictors were calculated using Cox proportional hazards methods. Logistic regression analysis was used only when the groups compared comprised more than 15 patients. The homogeneity of the groups compared in the OS analysis was confirmed using ANOVA, followed by a Bonferroni correction.

Cox regression was run with stepwise selection: predictors are added to the model when their significance level is below 0.05 (pe) and removed when it exceeds 0.10 (pr). The listed clinical variables were evaluated as potential predictors of the hazard. The goodness-of-fit of the models was assessed by comparing the log-likelihood values, Akaike information criterion (AIC) and Bayesian information criterion (BIC), with smaller AIC and BIC values suggesting a better fit.

In addition, we performed an ROC curve analysis, including area under the curve (AUC), to compare the predictive ability of the different classification systems, including aggressive vs. non-aggressive, FIGO 2009, FIGO 2023, and the new classification proposed by our group, considering all possible cutoff points across the different stages. The CI for AUC is calculated by the de Long method.

## 3. Results

### 3.1. Demographic Results

We collected data from 1181 patients from the three Spanish tertiary hospitals. The patients were divided into two main groups based on pathological features: 828 non-aggressive cases and 353 aggressive cases.

Demographic data, types of treatment applied, and staging according to the old and new 2023 FIGO staging systems are provided in Table 1, comparing aggressive and non-aggressive groups. Significant differences were found in age (older in the aggressive group), treatment, both classification FIGO systems, and stage changes between the previous and new FIGO staging systems that are more common in the aggressive tumors.

In the overall study, 202 women were re-staged under the new FIGO stage, while 979 cases (82.9%) required no changes in prognostic or treatment management (Table 1). The main finding was the statistically significant difference in stage shifts between the aggressive and non-aggressive groups, as well as among the five endometrial carcinoma subgroups (*p* < 0.001). Shifts occurred more frequently in the aggressive group compared to the non-aggressive group (43.9% vs. 5.7%), particularly upshifts (41.9% vs. 5.6%).

There were 148 cases that upshifted due to belonging to the aggressive histological subgroup itself, resulting in a change from Stage I in the 2009 FIGO staging to the new IIC stage. Another 45 cases upshifted due to substantial LVSI, from Stage I to Stage IIB. Downshifts were only detected in advanced-stage disease, from IVB to IIIB2, indicating metastasis to the pelvic peritoneum, observed in eight cases.

Regarding treatment, the inclusion criteria required all patients to undergo surgery. Among all patients, 249 cases (20.8%) received some form of postsurgical chemotherapy: 38 with parenteral chemotherapy, 72 with daily tamoxifen, 38 with GnRH analogs, and 101 with progestins. Postoperative radiotherapy was administered to 413 patients (34.7%): 135 cases (32.6%) with external radiation, 146 (35.4%) with brachytherapy, and 132 women (32%) with both external radiation and brachytherapy. Of the patients treated with radiotherapy, 26.9% also received chemotherapy, so 12.8% of all cases were treated with chemoradiotherapy.

There were 207 surgical complications: 65 were Clavien–Dindo grade I, 133 grade II, and 9 grade III. Only one case, with a major complication (Clavien–Dindo grade IV), was downstaged, indicating that only one case in the entire series could theoretically have avoided a severe complication. However, upon deeper examination, it was found to be a Stage IVB (FIGO 2009) shifted to a Stage IIIB2 according to the new FIGO classification, which would still require the same treatment and thus no change in the possibility of complications.

### 3.2. General Overall Survival

The median follow-up for the entire cohort was 42.6 months (IQR 23.6–60), and among patients with aggressive carcinoma, the mean OS was 36.2 months (IQR 14.4–60), and the median OS was 35.7 months. In contrast, patients with non-aggressive carcinoma had a median OS of 60 months (IQR 31.85–60).

### 3.3. Novel Classification

**Figure 2:**
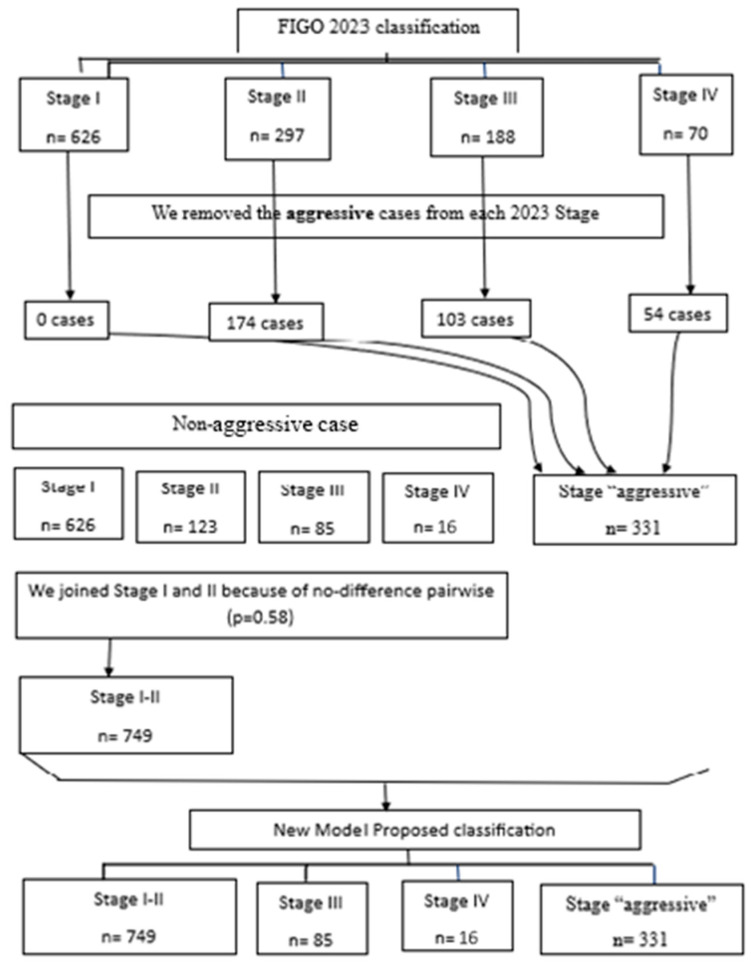
Flowchart of the design of the new proposed model.

Based on the preliminary results, we developed a novel classification derived from the FIGO 2023 classification. As illustrated in Figure 2, endometrial cancers were initially stratified into non-aggressive and aggressive categories, resulting in five groups: low-grade endometrial cancer Stages I, II, III, and IV, along with a distinct group comprising tumors with aggressive histological subtypes. Subsequently, Stages I and II within the non-aggressive category were combined, as no significant differences in survival outcomes were observed between them.

Subsequently, we conducted a comparative analysis of the following four models:

(a) ***In the first model,*** we only included the aggressive/non-aggressive classification, omitting the FIGO 2009 classification and the FIGO 2023 classification. When tumors were categorized as non-aggressive versus aggressive, the latter ones showed a significantly worse 5-year OS, with more than a twofold increased risk of death compared with non-aggressive tumors (OR = 2.51).

(b) ***In the second model,*** we only included the FIGO 2009 classification. According to the FIGO 2009 classification, Stage II was associated with a significantly lower risk of death, whereas Stages III and IV showed progressively higher odds of mortality, with Stage IV presenting the worst prognosis (OR = 2.71), but in all cases with significant results.

(c) ***In the third model,*** there was only included the FIGO 2023 classification. Using the FIGO 2023 classification, a clear stepwise deterioration in survival was observed with increasing stage. Compared with Stage I, Stages II, III, and IV were all associated with significantly higher odds of death, with the strongest effect seen in Stage IV.

(d) ***In the fourth model,*** our new model staging system was included alone. In the pairwise comparison between Stages I and II after exclusion of aggressive cases, no significant difference was observed (*p* = 0.58). Accordingly, both stages were combined into a new single Stage I. In the newly proposed stages, Stages I and II again served as the reference category.

#### Comparison Among Previous and New Classifications

- Table 2 shows the results of the frequency assessment of the five-year OS according to the different prognostic models. When tumors were categorized as non-aggressive versus aggressive, the latter ones showed a significantly worse five-year OS, with more than a twofold increased risk of death compared with non-aggressive tumors (OR = 2.51). According to the FIGO 2009 classification, Stage II was associated with a significantly lower risk of death, whereas Stages III and IV showed progressively higher odds of mortality, with Stage IV presenting the worst prognosis (OR = 2.71). Using the FIGO 2023 classification, a clear stepwise deterioration in survival was observed with increasing stage. Compared with Stage I, Stages II, III, and IV were all associated with significantly higher odds of death, with the strongest effect seen in Stage IV. In the new proposed classification, Stage I and II served as the reference, Stage III showed no significant enhancement of survival, while Stages IV and the new Stage “aggressive” were associated with markedly increased odds of death. The proposed model demonstrated a clear separation between low- and high-risk groups, particularly highlighting the adverse impact of advanced-stage disease.
- A series of stepwise Cox proportional hazards models, including all the variables collected, were performed to identify independent predictors of death at five years; results are shown in Table 3. This analysis aimed to evaluate the robustness of our hypothesis by means of a multivariate approach to the data.

**Figure 3:**
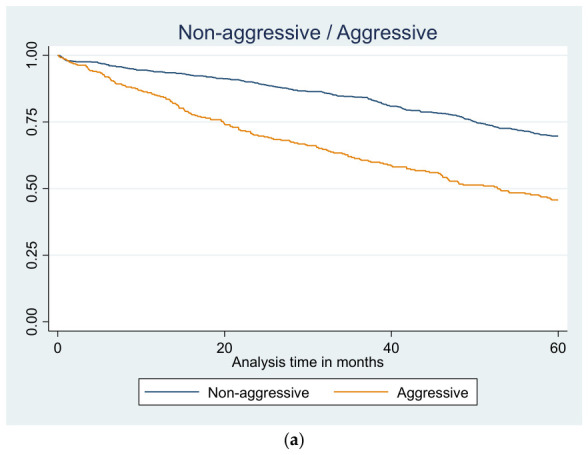

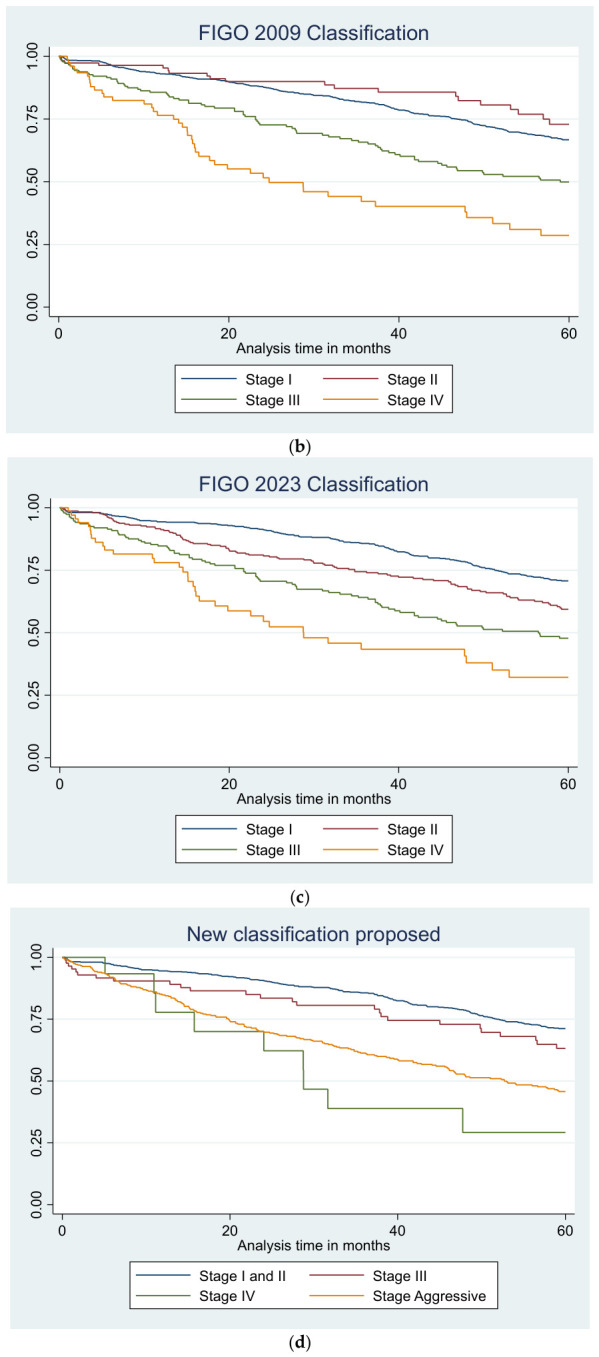
Overall survival Kaplan–Meier curves for the four different classification models. (**a**) First model comparing aggressive cases and non-aggressive cases. (**b**) Second model assessing the stages in FIGO 2009 classification. (**c**) Third model assessing the stages in FIGO 2023 classification. (**d**) Fourth model assessing the stages in the new classification proposed.

**Figure 4:**
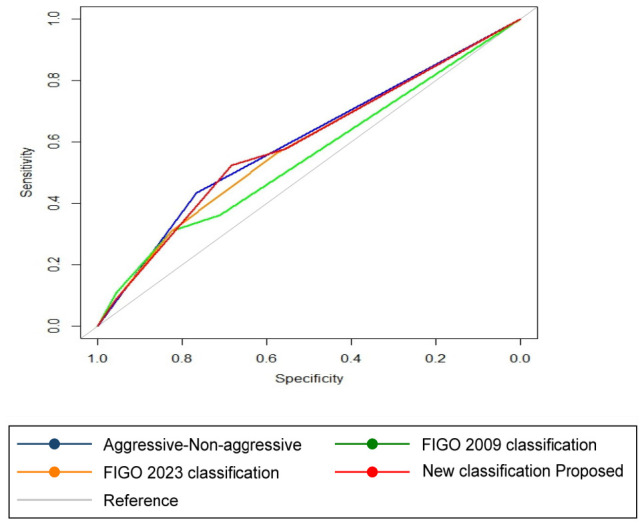
ROC curves comparing the four different models of endometrial cancer classification.

The binary variable aggressive/non-aggressive displayed a hazard ratio of 2.93 (95% IC: 2.01–4.28) itself. Assessing the FIGO 2009 classification alone, Stage II shows a significant HR of 2.85 (95% IC: 1.47–5.52); however, Stage III was associated with increased odds of death. This did not reach statistical significance, though the tendency leads to positive results, and Stage IV was not significant. According to the FIGO 2023 classification, Stage II has almost three times more risk of death than Stage I (HR 2.92, IQR: 1.66–5.13), Stage III has an HR of 2.21 (IQR: 1.14–4.26), and Stage IV is not statistically significant, with an HR of 1.39. About our new proposed model:

- Based on the results shown in Table 3, the hazard ratios of Stages III and “Stage aggressive” with respect to Stages I–II were significantly greater; Stage III has a hazard ratio of 2.34 and Stage “Aggressive” has an HR of 3.78. However, Stage IV’s HR is 1.1 with no significant result. When FIGO 2009 and FIGO 2023 classification variables were included, Stage II and Stage III—regardless of classification system—showed strong and significant associations with poorer outcomes. Depending on the staging scheme, Stage II and Stage III remained robust predictors, with hazard ratios ranging from 2.21 to 2.9. Stage IV did not consistently reach a statistically significant difference in HR compared to Stage I across either model.
- Across all model specifications, the likelihood-ratio tests were highly significant (*p* < 0.001). Variables consistently retained after stepwise selection included age, diabetes, myometrial invasion, LVSI, and adnexal involvement. They were independently associated with increased risk. Factors such as BMI, hypertension, cervical involvement, parity, treatment type, postoperative complications (Clavien–Dindo) and nodal status were repeatedly removed during stepwise selection, indicating no significant contribution to the model after adjustment.
- Kaplan–Meier survival curves showed a statistically significant difference in OS between aggressive and non-aggressive carcinoma groups, the FIGO 2009 classification, and the FIGO 2023 classification (*p* < 0.001). Our proposed staging system yielded statistically significant results (*p* < 0.001), providing further support for our hypothesis. Results are shown in Figure 3.
- As a final analysis, we calculated the area under the curve (AUC) for each of the different classification systems (non-aggressive/aggressive, FIGO 2009, FIGO 2023, and our newly proposed classification) and compared them to determine whether there were statistically significant differences (Figure 4 and Table 4). With these data, we can determine that the FIGO 2009 classification showed the lowest discriminatory performance. In contrast, the FIGO 2023 classification and the newly proposed model demonstrated comparable and superior performance. The aggressive/non-aggressive stratification was not significantly inferior to either the FIGO 2023 or the proposed model, suggesting similar predictive utility despite its simplicity.

## 4. Discussion

This study provides an in-depth analysis of survival differences and associated risk factors in patients with endometrial cancer. Our results demonstrate that overall survival (OS) in aggressive cases is significantly lower than in non-aggressive cases. This finding indicates that aggressive tumors differ substantially from non-aggressive ones in terms of biological behavior, treatment response, and prognosis, and therefore may require more individualized and intensive clinical management strategies. This concept has been previously suggested in the literature [3,18,19] and has already influenced recent changes in the FIGO classification, with greater weight being assigned to aggressive histological subtypes. However, our results extend this observation further, suggesting that the prognostic impact of aggressive histology should be even greater and all aggressive cases should be treated with adjuvant aggressive treatment. As shown by the hazard ratios (HRs) across the different models analyzed (dichotomous non-aggressive/aggressive model, FIGO 2009 classification, FIGO 2023 classification, and our newly proposed model), aggressive histology alone increases the risk of death by nearly threefold compared with non-aggressive tumors (HR 2.93; 95% CI 2.01–4.28).

Regarding the merging of low-grade (grades 1 and 2) non-aggressive carcinomas, Stages I and II, we considered that the survival curves were sufficiently close to justify grouping them into a single stage because of the pairwise results (*p* = 0.2). From a clinical perspective, it is reasonable to distinguish disease confined to the uterus and adnexa (with the nuances described in detail in the FIGO 2023 staging system) [4] from more extensive pelvic involvement or distant spread.

In the FIGO 2009 classification, the aggressiveness was not specifically considered; consequently, the hazard ratios across stages are neither consistently staggered nor uniformly statistically significant. The FIGO 2023 classification partially addresses this limitation, showing significant hazard ratios in some stages and an overall improvement in prognostic stratification. Nevertheless, the most pronounced improvement is observed with our new proposal stages, in which hazard ratios increase progressively across stages, except for low-grade endometrioid endometrial cancer Stage IV (HR 1.095; 95% CI 0.15–8.14; *p* = 0.93). This lack of statistical significance is likely attributable to the small number of Stage IV cases that had been removed from the aggressive cases (only 16 patients), which limits statistical power.

Among the four models evaluated, log-likelihood, AIC, and BIC values were broadly comparable, although slightly better for our proposed model. Importantly, our model demonstrated lower AIC and BIC values than the FIGO 2023 classification, indicating a better overall fit. Since both criteria penalize model complexity, these results suggest that our model achieves a more favorable balance between goodness of fit and parsimony. Overall, this indicates that although the models are similar in terms of validity, our proposal could provide a modest but meaningful improvement.

To further reinforce our hypothesis, we performed ROC curve analyses for each model. These analyses showed that the FIGO 2009 classification has insufficient discriminative ability, whereas both the FIGO 2023 classification and our proposed model demonstrate clearly superior predictive performance. Interestingly, the simplest model—based solely on the dichotomous distinction between aggressive and non-aggressive tumors—did not differ significantly in predictive capacity from either the FIGO 2023 classification or our proposed classification. This finding suggests that, in practical terms, simply distinguishing tumors by aggressive histology may be sufficient to classify patients with considerable prognostic accuracy, though obviously it could not be a real possibility.

Regarding demographic and clinical risk factors, both age and diabetes emerged as statistically significant predictors in the Cox regression analysis. Although these variables are often considered intuitive risk factors for mortality, several studies have demonstrated that age represents an independent and consistently relevant prognostic factor in oncology and should always be taken into account [20]. Other variables, such as myometrial invasion, LVSI, and adnexal involvement, also showed significant associations with survival. These factors are already recognized as independent prognostic variables and have been incorporated into the restaging of the FIGO 2023 classification. However, their hazard ratios were not sufficiently strong to justify a greater weighting in prognostic stratification. Interestingly, tumor grade did not influence outcomes when using the dichotomous aggressive/non-aggressive model, whereas it retained significance within the FIGO staging systems.

According to NCCN guidelines [21], patients upstaged from FIGO 2009 Stage I to FIGO 2023 Stage II may receive radiotherapy (external beam or brachytherapy) depending on tumor grade, whereas Stage II disease generally warrants external beam radiotherapy in all cases, with individualized brachytherapy and chemotherapy. Consequently, patients who were upstaged may have been undertreated, potentially explaining their poorer outcomes. This observation is consistent with findings reported by Schwameis et al. [22].

The ultimate goal of any staging or classification system is to improve patient care, survival, and disease-free intervals, thereby enhancing quality of life. From this perspective, it is crucial to consider which classification changes genuinely translate into treatment modifications. With the FIGO 2023 classification, many cases were upstaged from Stage I to Stage II solely due to aggressive histology: 74 cases (14.26%) in the Schwameis et al. [22] cohort, 73 patients (13.35%) in the study by Yu et al. [23], and 148 cases (12.53%) in our study. Although this upstaging increases the assigned risk group to a “high-risk group” in most cases, it does not always result in more aggressive treatment, particularly in the absence of comprehensive molecular profiling. Furthermore, according to the FIGO 2025 update [24], aggressive variants remain classified as “uncertain risk” even after complete molecular analysis, further complicating clinical management.

Our proposed classification aims to improve quality of care through a simple, pragmatic model that does not require extensive molecular testing, high financial investment, or advanced technological resources. This approach could be applied by clinicians in a wide range of healthcare settings, regardless of institutional complexity, facilitating clearer diagnostic stratification and more appropriate treatment planning [25]. The improved AUC observed with our model indicates enhanced prognostic performance, supporting the achievement of this objective. While current research trends favor increasingly complex, technology-driven personalized medicine, such approaches may not be feasible in all healthcare systems, potentially widening global disparities in cancer care Worldwide. Nowadays, only 48% of Spanish healthcare centers providing treatment for endometrial cancer have access to a comprehensive molecular analysis [26]. There are no global data available on this issue, except for certain highly developed countries [27]. However, if in a first-world country such as Spain only half of the centers are able to perform a complete molecular assessment, it is evident that it will be lower in less developed countries. On the other hand, there are not many countries that publish their molecular testing rates.

This study has several limitations. Molecular subtypes and estrogen receptor status could not be assessed due to insufficient case characterization, introducing potential bias, as these factors are now considered essential for risk stratification [28]. Additionally, the retrospective design and the inclusion of historical cases within our series limited the availability and granularity of certain variables, such as LVSI, which were not consistently recorded according to current standards [27].

## 5. Conclusions

Aggressive histological subtype in endometrial cancer should carry greater prognostic weight in terms of survival and clinical management. Our findings support a potential shift in the current paradigm for these relatively rare but high-risk cases, advocating for more aggressive treatment strategies to improve patient outcomes, although prospective studies are warranted, considering the clinical significance and potential impact of modifying treatment strategies in a disease as significant as endometrial cancer. Additionally, excluding aggressive cases, low-risk endometrial (G1-G2) cancer stages should be re-evaluated.

## Figures and Tables

**Table 1:** Patient characteristics were evaluated in the entire cohort and according to disease aggressiveness (non-aggressive versus aggressive cases).

	Total	Non-Aggressive	Aggressive	*p*
Number of patients	1181	828 (70.1%)	353 (29.9%)	
Age (years)	66.7 (57–74)	65.6 (57–72)	69.3 (60–77)	<0.001
BMI (Kg/m^2^)	31.6 (25.3–40)	31.8 (25.4–38.3)	31.3 (25–45.3)	0.35
Diabetes	239 (20.3%)	168 (20.3%)	71 (20.1%)	0.94
Hypertension	544 (45.9%)	376 (45.5%)	168 (46.8%)	0.69
Nulliparity	324 (27.5%)	230 (28%)	94 (27.5%)	0.6
				
**Treatment:**				<0.001
- Surgery only	522 (4.2%)	445 (3.7%)	77 (22.3%)	
- Surgery + Radiotherapy	413 (34.8%)	299 (36.2%)	114 (1.8%)	
- Surgery + Chemotherapy	94 (8%)	24 (2%)	70 (19.8%)	
- Surgery + Chemo-radiotherapy	152 (12.8%)	60 (7.3%)	92 (25.6%)	
				
**Stage FIGO 2009**				<0.001
I	811 (68.7%)	641 (77.4%)	170 (48.2%)	
II	112 (9.5%)	86 (10.4%)	26 (7.4%)	
III	179 (15.2%)	84 (10.2%)	95 (26.9%)	
IV	79 (6.7%)	17 (2.1%)	62 (17.6%)	
				
**New Stage FIGO 2023:**				<0.001
I	626 (53%)	604 (72.9%)	22 (6.2%)	
II	297 (25.1%)	123 (14.9%)	174 (49.3%)	
III	188 (15.9%)	85 (10.3%)	103 (29.2%)	
IV	70 (5.9%)	16 (1.9%)	54 (15.3%)	
				
**Change in Stage** from FIGO 2009 to FIGO 2023				0.000
- No shift	979 (82.9%)	781 (94.3%)	198 (56.1%)	
- Up-shift	194 (16.4%)	46 (5.6%)	148 (41.9%)	
- Down-shift	8 (0.7%)	1 (0.1%)	7 (2%)	

Data are given in median and 25th–75th percentile or frequencies and percentage.

**Table 2:** Frequency assessment of five-year overall survival according to the different models.

		OS 5 YEARS	OR	*p*
**Non-aggressive/Aggressive**	Non-aggressive	n = 608 (73.43%)	2.51 (1.94–3.25)	<0.001
	Aggressive	n = 185 (52.41%)		
**FIGO 2009 Classification**	Stage I	n = 563 (69.42%)	Ref.	
	Stage II	n = 92 (82.14%)	0.49 (0.3–0.82)	0.006
	Stage III	n = 102 (56.98%)	1.71 (1.23–2.39)	<0.001
	Stage IV	n = 36 (45.57%)	2.71 (1.7–4.33)	<0.001
**FIGO 2023 Classification**	Stage I	n = 457 (73%)	Ref.	
	Stage II	n = 198 (66.67%)	1.35 (1.00–1.82)	0.048
	Stage III	n = 103 (54.79%)	2.23 (1.59–3.13)	<0.001
	Stage IV	n = 35 (50%)	2.7041 (1.64–4.46)	<0.001
**New classification proposed**	Stage I and II	n = 727 (61.56%)	Ref.	
	Stage III	n = 85 (7.2%)	1.29 (0.79–2.11)	0.307
	Stage IV	n = 16 (1.35%)	3.77 (1.38–10.26)	0.009
	Stage “Aggressive”	n = 353 (29.89%)	2.66 (2.04–3.48)	<0.001

**Table 3:** Multivariable stepwise Cox regression analysis. Results are expressed as Hazard Ratio (95% CI) and *p*-value.

Variables	HR (95% CI) *	*p*-Value
Aggressive/non-aggressive	2.93 (2.01–4.28)	**<0.001**
FIGO 2009 Classification		
- Stage I	Ref	**-**
- Stage II	2.85 (1.47–5.52)	**0.002**
- Stage III	1.77 (0.94–3.33)	0.077
- Stage IV	0.74 (0.29–1.88)	0.527
FIGO 2023 Classification		
- Stage I	Ref	-
- Stage II	2.92 (1.66–5.13)	**<0.001**
- Stage III	2.21(1.14–4.26)	**0.018**
- Stage IV	1.39 (0.4–4.84)	0.604
New classification proposed		
- Stage I–II	Ref	-
- Stage III	2.34 (1.24–4.43)	**0.009**
- Stage IV	1.10 (0.15–8.14)	0.93
- Stage “aggressive”	3.78 (2.46–5.81)	**<0.001**

* Hazard ratio adjusted for all the variables collected. Only the classification model results were presented, though some additional variables yielded significant results: age, diabetes, adnexal invasion, myometrial invasion, LVSI, tumor grade and stage reclassification. The rest of the variables assessed were automatically removed from the model due to the lack of statistical significance.

**Table 4:** Comparison among the ROC curves of the different models of endometrial cancer classification (*p*-value).

	Aggressive/Non-Aggressive	FIGO 2009 Classification	FIGO 2023 Classification
FIGO 2009 classification	0.004		
FIGO 2023 Classification	0.37	<0.001	
Newly classification proposed	0.57	<0.001	0.29

## Data Availability

Due to confidentiality obligations, source privacy considerations, and ethical restrictions associated with the data collection process, the underlying dataset cannot be made publicly available. Sharing the data could compromise the anonymity and protection of the individuals and organizations involved, and would conflict with the ethical standards and agreements under which the data were obtained.

## Abbreviations

The following abbreviations are used in this manuscript:

FIGO	International Federation of Gynecology and Obstetrics
EC	Endometrial cancer
LVSI	Lymphovascular space invasion
OS	Overall Survival
OR	Odd Ratio
ROC	Receiver Operating Characteristic
CI	Confiance Interval
AUC	Area Under the Curve
IQR	Interquartile Range
HR	Hazard Ratio
